# Time-Specific Associations of Tumor Necrosis Factor-α Levels and Polymorphisms (−850 C/T or −308 G/A) With Suicidal Ideation in Acute Coronary Syndrome Patients

**DOI:** 10.3389/fpsyt.2021.739823

**Published:** 2021-09-23

**Authors:** Hee-Ju Kang, Ju-Wan Kim, Ju-Yeon Lee, Sung-Wan Kim, Il-Seon Shin, Young Joon Hong, Youngkeun Ahn, Myung-Ho Jeong, Jae-Min Kim

**Affiliations:** ^1^Department of Psychiatry, Chonnam National University Medical School, Gwangju, South Korea; ^2^Department of Cardiology, Chonnam National University Medical School, Gwangju, South Korea

**Keywords:** acute coronary syndrome, depression, tumor necrosis factor-α, gene association study, interaction

## Abstract

**Background:** Considering the association of inflammation with suicide and acute coronary syndrome (ACS), we investigated the individual and interactive effects of serum tumor necrosis factor-alpha (sTNFα) levels and two polymorphisms (−850 *C*/*T* and −308 *G*/*A*) on suicidal ideation (SI) after ACS.

**Methods:** The SI status using items on the Montgomery–Åsberg Depression Rating Scale (MADRS), related covariates including sociodemographic and clinical characteristics, sTNFα levels, and tumor necrosis factor-alpha (TNF-α) polymorphisms were evaluated in 969 patients within 2 weeks after ACS. Of the patients, 711 were evaluated 1 year later for SI. Multivariate logistic regression models were used to calculate individual and interactive associations after adjusting for the covariates.

**Results:** Higher (vs. lower) sTNFα levels and the −*850 C*/*T* or *T/T* (vs. C/C) polymorphism were significantly associated with SI 2 weeks after ACS, while only higher sTNFα levels were significantly associated with SI after 1 year. Significant interactive effects were detected between sTNFα (higher) levels and the −*850 C/T (C/C* or *C/T)* polymorphism on SI 2 weeks after ACS and between the two (−850 *CC* or *CT* and −*308 G/A* or *AA*) polymorphisms on SI 1 year after ACS.

**Conclusions:** The sTNFα level and two polymorphisms (−850C/*T* and −308 *G/A*), separately or in combination, could be time-specific biomarkers for SI in ACS. Focused interventions for ACS patients at risk of SI might reduce the suicidal burden in patients with ACS.

## Introduction

Suicide is a global health issue, and much effort has been dedicated to identify patients at risk of suicide and devise preventive strategies. However, no markers have been validated to predict SI, where suicide is a complex, multifactorial phenomenon involving interactions between various environmental stressors (e.g., childhood adversities and physical illness) and individual factors including hopelessness, temperament, and biological mechanisms [e.g., sensory processing, serotonergic system function, hypothalamic–pituitary–adrenergic (HPA) axis activity, inflammatory status, and genetic vulnerabilities] (1–4). Life-threatening physical illnesses, including acute coronary syndrome (ACS), are risk factors for suicide because of their psychological (e.g., hopelessness and psychiatric comorbidities) and biological (e.g., inflammation and a heightened stress response due to HPA axis activity) effects (5, 6). Previous epidemiological and clinical studies demonstrated an increased risk of suicide in ACS patients (6, 7). Suicide/suicidal behavior (SB) includes suicidal ideation (SI), suicidal attempts (SA), and death due to suicide. SI is associated with poor long-term cardiac outcomes in ACS patients (8). Therefore, it is important to investigate the pathophysiology and biological markers of suicide in ACS patients.

Previous studies reported that abnormal inflammatory responses, including changes in cytokines, contribute to SB, which includes SI and SA (9, 10). Additionally, inflammation is a well-established pathomechanism underlying ACS (11). Cytokines are key modulators of inflammation and promising biomarkers of SB in ACS patients (12). Tumor necrosis factor-alpha (TNF-α) is a proinflammatory cytokine produced by T cells, glial cells, and neurons. Tumor necrosis factor-alpha activates the serotonergic system of the brain by stimulating serotonin transport and decreasing extracellular serotonin (13). Accordingly, many studies have reported that an increase in the TNF-α level and expression is associated with SI, SA, and death (14–17). However, other studies have reported no such association (18, 19) or a negative association has been reported (20). For the studies of TNF-α polymorphism, TNF-α-308 G/A is associated with SA (21) and male suicide (22). However, others reported no such associations (23, 24).

Despite accumulating evidence for a high risk of suicidality in ACS patients (6, 7), and the associations between suicide and cytokine abnormalities (9, 10), no study has investigated the association between cytokine imbalance (including TNF-α) and suicidality in ACS patients. The TNF-α level is affected by TNF-α gene polymorphisms; −850T and −308A alleles result in higher TNF-α levels compared to −850C and −308G alleles (25, 26). Interventions focused on suicide prevention are needed for patients at high risk of SI within 2 weeks, and 1 year after, ACS onset, based on their TNF-α status (level and polymorphism). We hypothesized that increased inflammation within 2 weeks of ACS onset, evidenced by a higher TNF-α serum level or polymorphisms that increase TNF-α expression, may be associated with SI in ACS patients. Additionally, individual and interactive effects of TNF-α serum levels and polymorphisms on SI in ACS patients may vary according to the time elapsed since ACS. Therefore, we investigated the individual effects of the serum TNF-α level and polymorphisms (−850C/*T* and −308G/*A*), as well as their interactive effect, on SI within 2 weeks, and 1 year after, ACS onset.

## Materials and Methods

### Study Overview

**Figure 1:**
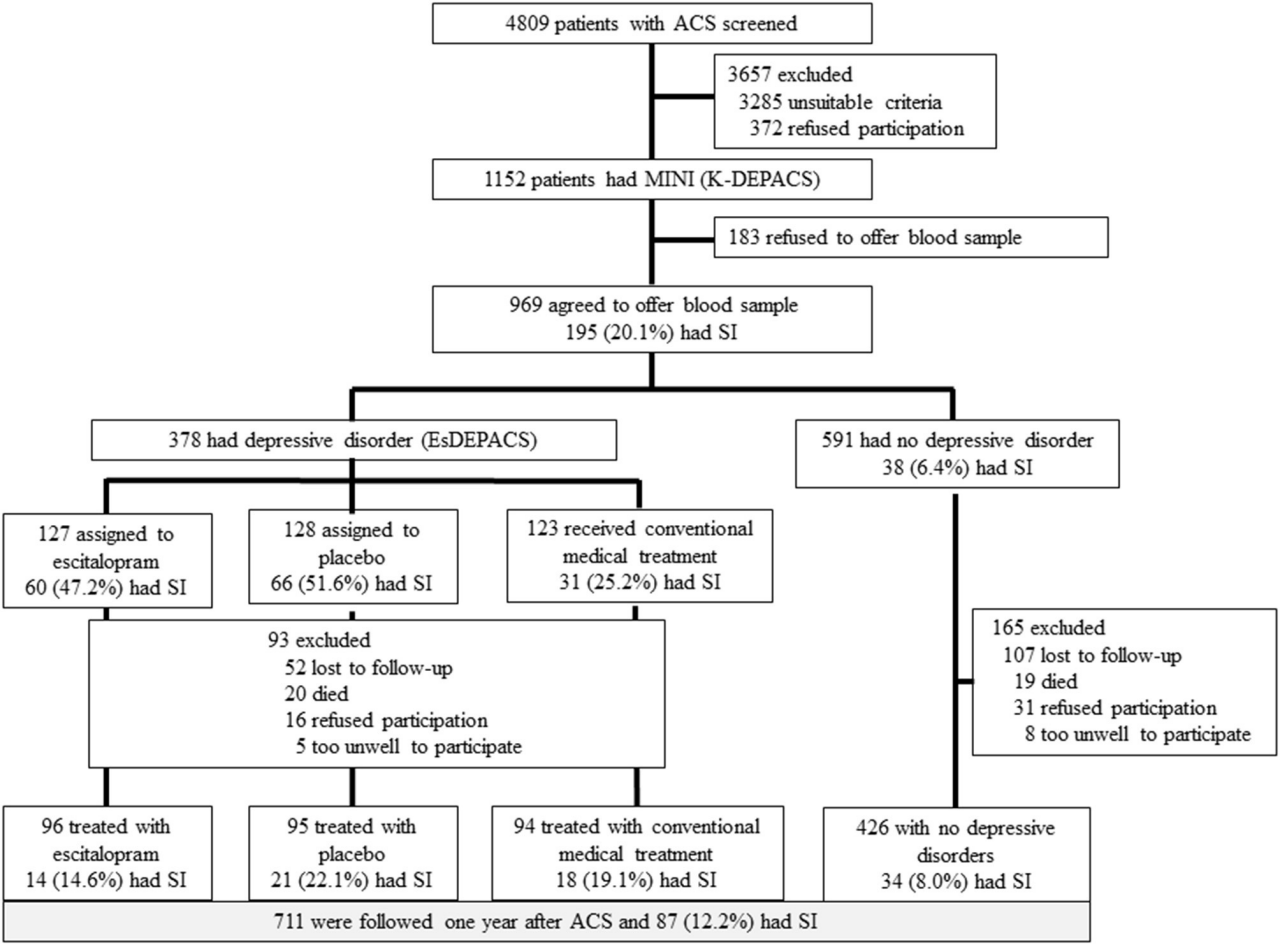
Flow diagram of the recruitment procedure. ACS, acute coronary syndrome; MINI, Mini-International Neuropsychiatric Interview; SI, suicidal ideation; K-DEPACS, Korean DEPression in Acute Coronary Syndrome study; EsDEPACS, Escitalopram for DEPression in Acute Coronary Syndrome study.

This study was a secondary analysis from an observational prospective study of Korean DEPression in ACS (K-DEPACS), which embraced part of a randomized controlled trial [Escitalopram for DEPression in ACS (EsDEPACS) study; ClinicalTrial.gov identifier: NCT00419471] (27). We recruited 1,152 consecutive ACS patients hospitalized at the Department of Cardiology, Chonnam National University Hospital, South Korea. Of these patients, 969 who met the inclusion criteria and consented to both participation and phlebotomy constituted the baseline sample (Figure 1). The K-DEPACS study included patients males and females aged 18–85 years, diagnosed with ACS [ST-segment elevation myocardial infarction (MI) was diagnosed on the basis of continuous chest pain ≥30 min, a new ST-segment elevation ≥2 mm on ≥2 contiguous electrocardiographic leads, and creatine kinase-MB (CK-MB) values ≥3 × normal limit; non-ST-segment elevation MI was diagnosed on the basis of chest pain and an increase in cardiac biochemical marker, without new ST-segment elevation; and unstable angina was diagnosed on the basis of chest pain within the last 72 h with or without ST-T segment changes or an increase in cardiac biochemical markers] and able to complete the questionnaires, understand the objectives of the study, and provide written, informed consent. Patients with any of following were excluded from K-DEPACS: ACS during hospitalization for another indication, ACS within 3 months after a coronary artery bypass graft surgery, uncontrolled hypertension [systolic blood pressure (BP) >180 mmHg or diastolic BP >100 mmHg], resting heart rate <40/min, severe medical disorders that are life threatening or may interfere with ACS recovery, or clinically significant laboratory abnormalities. Additional inclusion criteria for EsDEPACS included a Beck Depression Inventory (28) score >10 and a major or minor depressive disorder diagnosed on the basis of the DSM-IV. Additional exclusion criteria for EsDEPACS were concurrent use of class I antiarrhythmic medications, reserpine, guanethidine, clonidine, methyldopa, lithium, anticonvulsants, antipsychotics, or antidepressants; previously diagnosed neuropsychiatric disorders such as dementia, Parkinson's disease, brain tumor, psychosis, bipolar disorder, alcoholism, or other substance dependence; pregnancy; and inclusion in ongoing trials of another drug.

Baseline assessments of SI and sociodemographic characteristics, cardiovascular risk factors, and current cardiac status (as described in section “Evaluation of SI and Covariates” in section “Materials and Methods”) were made within 2 weeks (mean = 6.3 ± 2.4 days) after ACS; 711 patients were evaluated at 1 year after ACS onset. The Chonnam National University Hospital Institutional Review Board approved K-DEPACS and EsDEPACS, and all subjects provided written informed consent.

### Evaluation of SI and Covariates

SI was measured within 2 weeks of the onset of ACS and after 1 year, using the suicide-related items of the Montgomery–Åsberg Depression Rating Scale (MADRS) (29). The MADRS consists of 10 items (total score range: 0–60). For the assessment of suicide-related items, participants were asked whether they thought life was worth living and whether they had ever planned a suicide attempt. Scores on the items ranged from 0 (life satisfaction) to 6 (explicit plans for suicide). The SI was defined by a score of 2 (fleeting suicidal thoughts) or more, as described previously (30). Although the suicide-related item of MADRS used in this study is a component of the depression rating scale, it was used in previous clinical trials and was found to be sensitive to changes in SI (30).

Data on the covariates strongly associated with SI in ACS patients (31) were evaluated within 2 weeks after ACS: (i) sociodemographic and depression characteristics including the age, sex, years of education, living status (living alone or not), type of residence (owned or rented), current occupation (employed or not), personal and family histories of depression, and depression diagnosis and treatment allocation status (escitalopram, placebo, treatment as usual); (ii) cardiovascular risk factors, including personal and family history of ACS, diagnosed hypertension and diabetes mellitus, hypercholesterolemia (fasting serum total cholesterol level >200 mg/dl or ongoing treatment), obesity (body mass index >25 kg/m^2^), and reported current smoking status and statin use; and (iii) the current cardiac status including ACS severity according to the Killip classification (32), left ventricular ejection fraction (echocardiography), heart rate (electrocardiography), and serum cardiac biomarkers (troponin I and CK-MB).

Depression was diagnosed on the basis of the DSM-IV criteria using the structured Mini International Neuropsychiatric Interview (33), within 2 weeks of ACS onset and after 1 year. The Killip classification is a simple clinical tool (classes I–IV) to stratify ACS patients according to the risk of poor clinical outcomes (34). Cardiologists who participated in our study and were blind to the depression status of participants determined the Killip classification.

### Serum TNF-α and TNF-α Polymorphism

Venous blood from participants in a fasting state was collected and used for assays in present analyses. Serum tumor necrosis factor-alpha (sTNFα) levels were evaluated using a solid-phase sandwich enzyme-linked immunosorbent assay kit (Invitrogen, Camarillo, CA, USA), and the TNF-α polymorphisms were analyzed using polymerase chain reaction (PCR) and PCR-based restriction fragment length polymorphism assays. Polymorphism selection and allele detection methods are described in Supplementary Table 1. Serum tumor necrosis factor-alpha levels were categorized into lower and higher groups using the median values. The TNF-α −850*C/T* and −308*G/A* polymorphisms were classified into two groups, “*C/C*” and (“*C/T*” or “*T/T*”) and “*G/G*” and (“*G/A*” or “*A/A*”), considering their infrequency.

### Statistical Analyses

Baseline characteristics were compared between patients with and without SI within 2 weeks of ACS onset, and after 1 year, using Student's *t*-tests or χ^2^-tests. Since the factors that affect SI during the acute and chronic phases might differ (35, 36), SI within 2 weeks of ACS onset and after 1 year were considered as separate dependent variables. Significant factors of SI within 2 weeks and 1 year after ACS onset (*p* < 0.05) were included as separate covariates in the multivariate analyses.

To evaluate the associations between sTNFα levels and polymorphisms, sTNFα levels were compared between patients with and without SI using Student's *t*-tests. A multivariate logistic regression model adjusted for relevant covariates was used to test the individual effects of sTNFα levels and polymorphisms on SI. To test for interactive effects between sTNFα levels and polymorphisms, participants were stratified based on their polymorphisms status (−850*C/T* and −308*G/A*). Then, the associations between sTNFα level and SI status were calculated using the same multivariate logistic regression models after adjusting for relevant covariates. To test for the interactive effects between two polymorphisms, participants were stratified by the *TNF-*α −*850 C/T* polymorphism status, and then the associations between *TNF-*α −*308 G/A* and SI status were analyzed using the same multivariate logistic regression models after adjusting for relevant covariates. The SPSS 21.0 software (SPSS Inc., Chicago, IL, USA) was used for all analyses.

## Results

### Baseline Characteristics and Recruitment

All patients (*N* = 4,809) admitted with a recent ACS were approached for study participation. Among these patients, 1,152 were eligible for inclusion in the study and agreed to participate. Of these, 969 participants who agreed to blood/genetic testing were included in the present analyses. No significant differences were observed in baseline characteristics between those who did and those who did not undergo blood/genetic test. Of the 969 patients, 711 (73%) were followed for 1 year. The 258 patients lost to follow-up were older and had a higher Killip lass (*p*-value < 0.05). SI was identified in 195 (20.1%) and 87 (12.2%) patients within 2 weeks of ACS onset and after 1 year, respectively. The baseline characteristics associated with SI within 2 weeks of ACS onset and after 1 year were considered covariates (Supplementary Table 2).

### Individual Effects of sTNFα Level and the TNF-α Polymorphisms on SI Status

Serum tumor necrosis factor-alpha levels were significantly higher in ACS patients with a higher frequency of the −850T and −308A alleles (all *P*-values < 0.05, Supplementary Figure 1). Table 1 summarizes the individual effects of the sTNFα level and the polymorphisms on SI. SI within 2 weeks after ACS was associated with a higher sTNFα level at baseline and a higher frequency of *TNF-*α −*850 C*/*T or T/T* alleles after adjusting for covariates. The significance of the association with the TNF-α –308 G/A allele was lost after adjustment. SI at 1 year after ACS was associated with a higher baseline sTNFα level, while no association was found with any of the TNF-α polymorphisms. All polymorphisms were in Hardy–Weinberg equilibrium (all *P* > 0.05).

**Table 1:** Individual associations of serum tumor necrosis factor-alpha (sTNFα) level and two TNF-α polymorphisms with SI.

**Exposure**	**Group**	***N***	**SI at baseline (*****n*** **= 969)**			***N***	**SI at follow-up (*****N*** **= 711)**		
			**No. (%) present**	**OR (95% CI)**			**No. (%) present**	**OR (95% CI)**	
				**Unadjusted**	**Adjusted^a^**			**Unadjusted**	**Adjusted^b^**
sTNFα	Lower	484	73 (15.0)	1.00	1.00	355	21 (5.9)	1.00	1.00
	Higher	485	122 (25.2)	1.89 (1.37–2.61)^‡^	1.75(1.24–2.48)^†^	356	66 (18.5)	3.62 (2.16–6.06)^‡^	3.40(2.01–5.75)^‡^
*TNF-α* –*850C/T*	C/C	715	135(18.9)	1.00	1.00	526	64 (12.2)	1.00	1.00
	C/T or T/T	254	60 (23.6)	1.33 (0.94–1.88)	1.49 (1.03–2.15)^*^	185	23 (12.4)	1.03 (0.62–1.71)	1.10 (0.65–1.85)
*TNF-α* –*308G/A*	G/G	781	146 (18.7)	1.00	1.00	577	69 (12.0)	1.00	1.00
	G/A or A/A	188	49 (26.1)	1.53 (1.06–2.22)^*^	1.27 (0.85–1.91)	134	18 (13.4)	1.14 (0.66–1.99)	1.02 (0.57–1.81)

a *Adjusted for gender, education level, housing, current employment status, personal depression history, statin use, and depression diagnosis and treatment allocation status at baseline*.

b *Adjusted for gender, family depression history, statin use, and depression diagnosis and treatment allocation status*.

* *P < 0.05;*

† *P < 0.01;*

‡ *P < 0.001*.

### Interactive Effects of sTNFα Level and TNF-α Polymorphisms According to SI Status

The sTNFα levels (continuous variables) were compared by SI status for each *TNF-*α polymorphism (Table 2). SI within 2 weeks after ACS was significantly associated with a higher sTNFα level only in ACS patients with the −850 C/*T* or *T/T* polymorphism, while SI at 1 year after ACS was correlated with a higher sTNFα level only in ACS patients with the –*308 G/G* genotype.

**Table 2:** Tumor necrosis factor-α (TNF-α) mean (SD) pg/ml serum concentrations by TNF-α two polymorphisms and depressive disorder status at 2 weeks and at 1 year after acute coronary syndrome (ACS).

		**SI at 2 weeks after ACS**			**SI at 1 year after ACS**		
		**Absent** **(*N* = 774)**	**Present** **(*****N*** **= 195)**	***p*** **-value**	**Absent** **(*****N*** **= 624)**	**Present** **(*****N*** **= 87)**	***p*** **-value**
*TNF-α* –*850C/T*	C/C	52.2 (37.8)	50.2 (10.9)	0.652	51.9 (34.9)	57.9 (23.5)	0.178
Genotype, *N* (%)	C/T or T/T	50.7 (13.9)	62.8 (57.0)	0.007	51.6 (13.9)	55.7 (9.0)	0.179
*TNF-α* –*308G/A*	G/G	51.3 (27.2)	55.7 (40.5)	0.114	51.2 (28.6)	58.9 (22.5)	0.032
Genotype, *N* (%)	G/A or A/A	53.4 (36.3)	61.1 (44.3)	0.234	54.7 (39.0)	51.5 (9.0)	0.728

*p-value using t-tests*.

**Figure 2:**
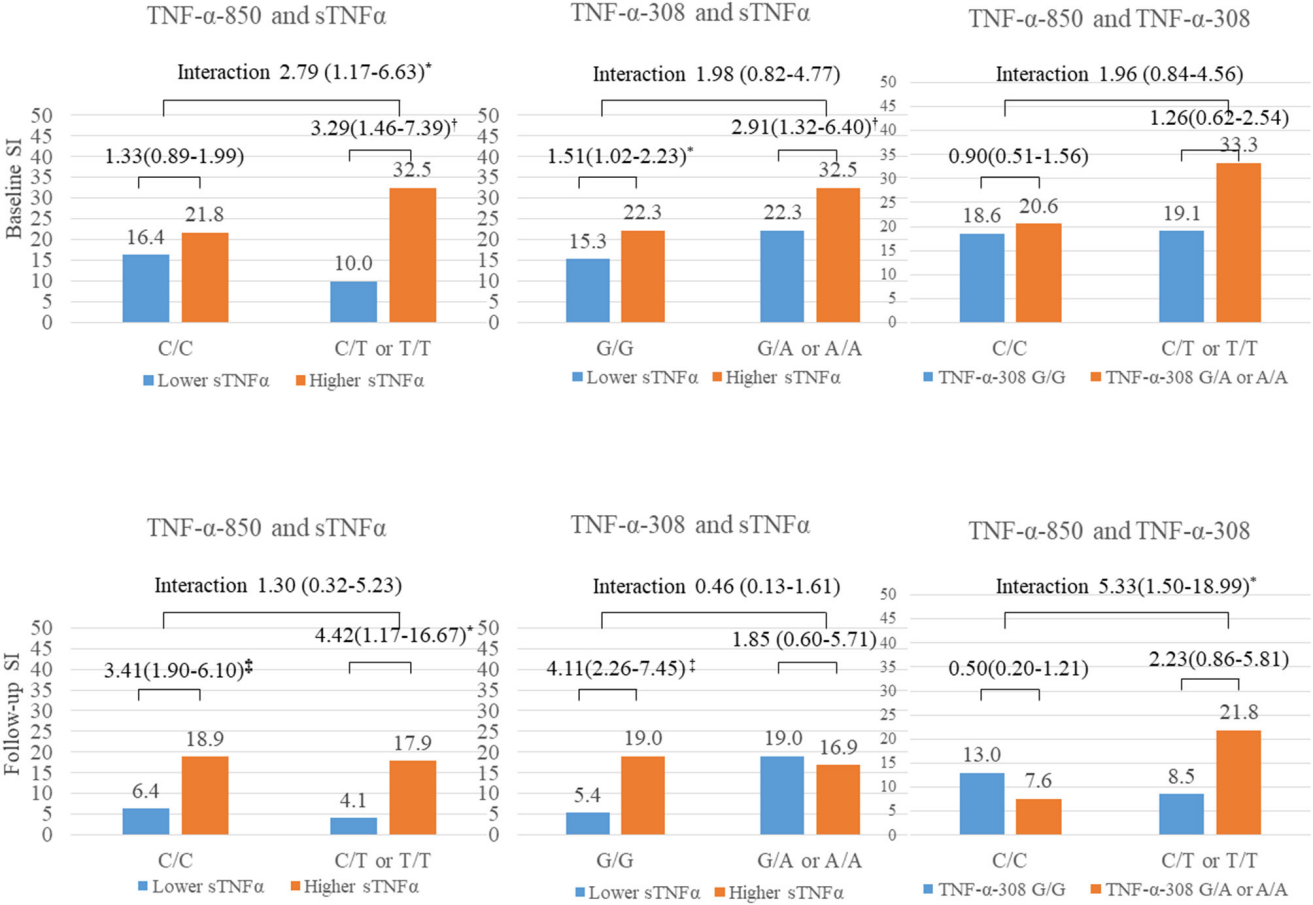
Interactive effect of tumor necrosis factor-alpha (sTNFα) level and two polymorphisms on suicidal ideation (SI) 2 weeks and 1 year after acute coronary syndrome (ACS). TNF-α, tumor necrosis factor-alpha; sTNFα, serum TNF-α level; SI, suicidal ideation. *P < 0.05; *P* < 0.01; *P* < 0.001.

The interactive effect between the sTNFα level (binary variable) and the TNF-α polymorphisms are described in Figure 2. SI at baseline was significantly associated with a higher sTNFα level in the presence of the −850 C/*T* or *T/T* genotype after adjusting for covariates. SI at 1 year after ACS was significantly associated with a higher sTNFα level in the presence of the –*308 G/G* genotype after adjustment, but no significant interaction was found. Instead, significant interactions were found between the two (−850 *CC* or *CT* and –*308 G/A* or *AA*) polymorphisms on SI at 1 year after ACS.

## Discussion

The main findings of this 1-year longitudinal study of ACS patients were that SI during the acute phase of ACS was significantly associated with higher baseline sTNFα levels and the −*850* C/*T* or *T/T* polymorphism, both individually and interactively. SI during the chronic phase of ACS was associated with higher sTNFα levels individually and significantly interacted with the two polymorphisms. SI is an early and mild form of SB. However, a study including participants from several countries reported that 60% of patients transition from SI to a SA during the first year after SI onset (37). Therefore, it is essential to detect SI in the early phase after onset of severe physical illness to prevent SA.

In this study, higher sTNFα levels within 2 weeks after ACS were associated with SI, both within 2 weeks and after 1 year. This suggests that an increase in TNF-α immediately after ACS plays a role in the pathophysiology of SI in ACS patients. The association between TNF-α and suicide has not been confirmed in the psychiatric or general population. Higher TNF-α levels are associated with SA [plasma, (14)], suicidal death [brain, (15, 16)], and SI in depressed patients [serum, (17)]. However, TNF-α levels are not associated with SI, SA, or death [CSF and plasma, (19); plasma, (18)], whereas decreased TNF-α levels have been associated with SI in depressed adolescents [plasma, (20)]. These inconsistent findings may be due to differences in study design, including suicide-related outcomes, study participants, and the bio-samples measured. Moreover, no longitudinal study has been performed on the association between TNF-α level and later SI, except the antidepressant response on changes in SI (17, 38). The heightened inflammatory status of the sTNFα level following ACS (11) may have contributed to susceptibility to SI in response to a stressful event such as ACS through an interaction with the serotonergic system and neuroplasticity (39, 40).

It was interesting that the interactive effect of the TNF-α polymorphisms and sTNFα level on SI differed by time elapsed after ACS was diagnosed. The TNF-α −*850* C/*T* polymorphism was associated with SI within 2 weeks after ACS individually and interactively with a higher sTNFα level. The significant interaction means that the association between a higher sTNFα level and SI within 2 weeks after ACS was prominent in the presence of the risk allele (−850 T). This inherited vulnerability, particularly the TNF-α −850 *C/T* or *T/T* polymorphism, which is associated with a higher sTNFα level (25), may potentiate the deleterious effect of an increased sTNFα level in response to ACS (11, 41). This proinflammatory state contributed to patients who faced the life-threatening event of ACS being susceptible to SI even in the acute phase of ACS. Although there is a lack of previous studies on the association between the TNF-α −*850* C/*T* polymorphism and suicide ranging from SI to SA, our findings provide evidence for inflammatory dysregulation underlying SB and provide insight for future studies investigating the association between TNF-α and suicide to consider the interaction between the sTNFα level and these polymorphisms.

No interactions were detected between the sTNFα level and individual polymorphisms on SI at 1 year after ACS, but a significant interaction was identified between the two polymorphisms and SI. During the chronic phase of ACS when adaptation to the changes after ACS has occurred, the interactive effect of the two TNF-α genetic polymorphisms rather than a single inherited polymorphism may have contributed to ACS patients experiencing SI. Although no previous studies have considered the interaction between the two TNF-α polymorphisms and underlying mechanisms, future research with larger samples is needed to ascertain these time-specific and synergistic genetic associations underlying SI in ACS patients.

This study had several strengths. It was the first longitudinal study to investigate the associations between TNF-α measured within 2 weeks and SI within 2 weeks, and at 1 year after, ACS onset. Additionally, this was the first study to investigate the individual and interactive effects of sTNFα levels and two polymorphisms (−850 *C*/*T* and −308 *G*/*A*) on SI. Moreover, many clinical and psychosocial covariates were assessed using validated instruments for psychiatric and cardiovascular evaluations. All consecutive eligible patients with recent ACS were recruited and followed up after 1 year, which reduced the error due to discordant examination intervals and increased sample homogeneity.

There were several limitations to this study. First, SI was measured using suicide-related items on a depression scale, rather than by a dedicated psychometric instrument (such as the Columbia Classification Algorithm of Suicide Assessment) (42). Although the suicide-related items of the MADRS are sensitive to changes in SI (30), future studies using scales (e.g., Columbia Classification Algorithm of Suicide Assessment) that are more specific and sensitive to SI assessment are needed to confirm our findings. Second, our findings on SI may not apply to SB in general in patients with ACS. Nevertheless, SI alone imposes a considerable healthcare burden (43) and can lead to severe SB (44). Third, the frequent co-occurrence of SI and depression needs further evaluation. In this study, 38 (80.5%) of 195 ACS patients had SI within 2 weeks of ACS onset, while 77 (88.5%) of 87 ACS patients with SI had depression at 1 year after ACS. SI is significantly associated with depression. In fact, it is one of the diagnostic criteria for depression (45). Previous studies have reported unique, shared, and interactive pathophysiological mechanisms underlying depression and suicide (46, 47). Inflammatory dysregulation has also been suggested as an underlying mechanism of depression, particularly in ACS patients during the acute phase of illness (48). In this study, the relationship between SI and TNFα levels/polymorphisms remained significant despite adjusting for depression status/treatment as a confounding factor. However, the association of SI with depression should be considered when interpreting the results of our study. Fourth, sTNFα levels were measured only once (at baseline); therefore, longitudinal associations between changes in sTNFα levels and SI could not be evaluated. Also, this study included a relatively small sample. However, polymorphism studies require large sample sizes. Moreover, we did not evaluate mental disorder history, including anxiety disorders, which may affect SI and should be considered as a potential covariate. Additionally, healthy controls were not included in this study, which precluded comparison of sTNFα levels between ACS patients and healthy controls. Future studies including healthy controls will be needed. Finally, attrition should be considered when interpreting the results. Of the participants, 84% agreed to undergo blood/genetic testing, and 73% of these patients were followed up. There was no difference between patients who did and did not undergo blood/genetic testing, but patients lost to follow-up were more likely to be older and have worse cardiac function.

In conclusion, a significant individual and interactive effect of sTNFα levels and the −850 *C/T* polymorphism on SI within 2 weeks after ACS onset was identified. A significant individual effect of the sTNFα level and interactive effects of the two TNF-α polymorphisms on SI at 1 year after ACS onset were observed. Our findings suggest that sTNFα levels and polymorphisms (−850 *C*/*T* and −308 *G*/*A*), individually or in combination, could be used as time-specific biomarkers of SI in ACS patients. In other words, measuring the sTNFα levels within 2 weeks after ACS and/or *850* C/*T* polymorphism can identify ACS patients at high risk of SI during the acute phase of the illness. Measuring the sTNFα levels or both polymorphisms (−850 *C/T* and –*308 G/A*) within 2 weeks can identify ACS patients at high risk for SI in the chronic phase of the illness who require preventive management. Detection of SI in ACS patients is crucial because SI is associated with poor long-term cardiac outcomes in ACS patients (8). Evaluation of sTNFα levels and polymorphisms in ACS patients may help clinicians to identify vulnerable patients who may benefit from focused, collaborative, and preventive strategies for SI. Our result will provide a basis for future research on the role of TNF-α in SI in ACS patients. Long-term clinical outcome studies are required on the effects of early identification of SI and preventive management in ACS patients. Further studies are warranted to evaluate the reproducibility of our results and cost-effectiveness of testing for sTNFα levels and polymorphisms.

## Data Availability Statement

The raw data supporting the conclusions of this article will be made available by the inquries to the corresponding author.

## Ethics Statement

The studies involving human participants were reviewed and approved by The Chonnam National University Hospital Institutional Review Board. The patients/participants provided their written informed consent to participate in this study.

## Author Contributions

H-JK and J-MK conducted the data analysis and drafted the article. J-WK, J-YL, S-WK, and I-SS helped to analyze the data and to draft the article. YH, YA, and M-HJ helped to recruit the participants and perform cardiac assessment and management. All authors contributed the article and approved the submitted version.

## Funding

The study was funded by a grant of the National Research Foundation of Korea Grant (NRF-2020M3E5D9080733 and NRF-2020R1A2C2003472) to J-MK.

## Conflict of Interest

The authors declare that the research was conducted in the absence of any commercial or financial relationships that could be construed as a potential conflict of interest.

## Publisher's Note

All claims expressed in this article are solely those of the authors and do not necessarily represent those of their affiliated organizations, or those of the publisher, the editors and the reviewers. Any product that may be evaluated in this article, or claim that may be made by its manufacturer, is not guaranteed or endorsed by the publisher.
