# Supplementary material for: Time-Specific Associations of Tumor Necrosis Factor-α Levels and Polymorphisms (−850 C/T or −308 G/A) With Suicidal Ideation in Acute Coronary Syndrome Patients
Source: Front Psychiatry. 2021 Sep 23;12:739823. doi: 10.3389/fpsyt.2021.739823 (PMC8496737; doi:10.3389/fpsyt.2021.739823)

**Time-specific associations of tumor necrosis factor-α levels and polymorphisms (-850 C/T or -308 G/A) with suicidal ideation in acute coronary syndrome patients**

**Hee-Ju Kang^a^, Ju-Wan Kim^a^, Ju-Yeon Lee, Sung-Wan Kim^a^, Il-Seon Shin^a^, Young Joon Hong^b^, Youngkeun Ahn^b^, Myung Ho Jeong^b^, Jae-Min Kim^a^***

***Supplementary information***

**SUPPLEMENTARY TABLE S1….……..…………………… ………….………………..2**

**SUPPLEMENTARY TABLE S2.……………….…………………... ……………………..4**

**SUPPLEMENTARY FIGURE S1….……..………………………………………………..7**

| **Supplemental Table 1** Polymerase chain reaction (PCR) methods for allele detection | | | | | | |
| --- | --- | --- | --- | --- | --- | --- |
| **Polymorphism** | **Forward (F) and reverse (R) primer** | **Restriction enzyme** | **Allele** | **Allele Frequency** | | |
|  |  |  |  | **Present study** | **East Asian** | **Caucasian** |
| TNF-ɑ -850C/T | F: 5’-TCGAGTATCGGGGACCCCCCGTT-3’ | *Hinc* II | -850*C* | 0.86 | 0.87 | 0.75 |
|  | R: 5’-CCAGTGTGTGGCCATATCTTCTT-3’ |  | -850*T* | 0.14 | 0.13[1] | 0.25 [2] |
| TNF-ɑ -308G/A | F: 5’-AGGCAATAGGTTTTGAGGGCCAT-3’ | *Nco* I | -308*G* | 0.90 | 0.98 | 0.85 |
|  | R: 5’-TCCTCCCTGCTCCGATTCCG-3’ |  | -308*A* | 0.10 | 0.02 [3] | 0.15 [4] |
|  | | | |  |  |  |

Reference for Supplementary Table 1

1. Zhao, Y., Xia, S., Zou, L., 2007. The Association between Polymorphism of TNF-α Gene and Hypertensive Disorder Complicating Pregnancy. J Huazhong Univ Sci and Technolog Med Sci. 27, 729-732.
2. McCusker, S.M., Curran, M.D., Dynan, K.B., McCyllagh, C.D., Urquhart, D.D., Middleton, D., Patterson, C.C., McIlroy, S.P., Passmore, A.P., 2001. Association between polymorphism in regulatory region of gene encoding tumour necrosis factor alpha and risk of Alzheimer's disease and vascular dementia: a case-control study. Lancet. 357, 436–439
3. Higuchi, T., Seki, N., Kamizono, S., Yamada, A., Kimura, A., Kato, H., Itoh, K., 1998. Polymorphism of the 5’-flanking region of the human tumor necrosis factor (TNF)-a gene in Japanese. Tissue Antigens. 51, 605-612
4. Misener, V.L., Gomez, L., Wigg, K.G., Luca, P., King, N., Kiss, E., Daróczi, G., Kapornai, K., Tamas, Z., Mayer, L., Gádoros, J., Baji, I., Kennedy, J.L., Kovacs, M., Vetró, A., Barr, C.L.; International Consortium for Childhood-Onset Mood Disorders., 2008. Cytokine genes TNF , IL1A , IL1B , IL6 , IL1RN and IL10, and childhood-onset mood disorders. Neuropsychobiol. 58, 71-80.

| **Supplementary Table 2.** Baseline sociodemographic and clinical characteristics by suicidal ideation (SI) status | | | | | | | |
| --- | --- | --- | --- | --- | --- | --- | --- |
|  | Baseline sample (N = 969) | | |  | Follow-up sample (N = 711) | | |
|  | No SI  (N = 774) | SI  (N = 195) | p-value |  | No SI  (N = 624) | SI  (N = 87) | p-value |
| **Sociodemographic characteristics** |  |  |  |  |  |  |  |
| Age, mean (SD) years | 58.0 (11.3) | 58.9 (10.6) | 0.315 |  | 57.6 (10.7) | 57.4 (11.1) | 0.855 |
| Sex, N (%) female | 201 (26.0) | 68 (34.9) | **0.013** |  | 163 (26.1) | 33 (37.9) | **0.021** |
| Education, mean (SD) year | 10.0 (4.7) | 9.1 (4.4) | **0.012** |  | 10.0 (4.6) | 9.2 (4.8) | 0.118 |
| Living alone, N (%) yes | 71 (9.2) | 21 (10.8) | 0.497 |  | 49 (7.9) | 11 (12.6) | 0.132 |
| Housing, N (%) rented | 109 (14.1) | 41 (21.0) | **0.017** |  | 102 (16.3) | 20 (23.0) | 0.124 |
| Currently unemployed, N (%) | 279 (36.0) | 89 (45.6) | **0.014** |  | 213 (34.1) | 38 (43.7) | 0.081 |
| **Depression characteristics** |  |  |  |  |  |  |  |
| Personal history of depression, N (%) | 19 (2.5) | 15 (7.7) | **< 0.001** |  | 24 (3.8) | 6 (6.9) | 0.247 |
| Family history of depression, N (%) | 15 (1.9) | 8 (4.1) | 0.108 |  | 14 (2.2) | 6 (6.9) | **0.026** |
|  |  |  |  |  |  |  |  |
| **Cardiac risk factors**, N (%) |  |  |  |  |  |  |  |
| Personal history of ACS | 30 (3.9) | 9 (4.6) | 0.639 |  | 25 (4.0) | 6 (6.9) | 0.255 |
| Family history of ACS | 24 (3.1) | 7 (3.6) | 0.729 |  | 18 (2.9) | 6 (6.9) | 0.103 |
| Hypertension | 360 (46.5) | 98 (50.3) | 0.349 |  | 282 (45.2) | 42 (48.3) | 0.588 |
| Diabetes mellitus | 144 (18.6) | 47 (24.1) | 0.085 |  | 118 (18.9) | 24 (27.6) | 0.058 |
| Hypercholesterolemia | 384 (49.6) | 102 (52.3) | 0.501 |  | 333 (53.4) | 50 (57.5) | 0.472 |
| Obesity | 341 (44.1) | 74 (37.9) | 0.123 |  | 280 (44.9) | 35 (40.2) | 0.414 |
| Current smoker | 297 (38.4) | 69 (35.4) | 0.442 |  | 247 (39.6) | 33 (37.9) | 0.768 |
| Statin use | 583 (75.3) | 129 (66.2) | 0.010 |  | 473 (75.8)_ | 52 (59.8) | 0.001 |
| **Current cardiac status** |  |  |  |  |  |  |  |
| Killip class >1, N (%) | 132 (17.1) | 36 (18.5) | 0.643 |  | 101 (16.2) | 13 (14.9) | 0.767 |
| LVEF, mean (SD) % | 61.2 (11.4) | 61.1 (10.8) | 0.911 |  | 61.4 (11.0) | 59.7 (11.8) | 0.183 |
| Heart rate, mean (SD) beat/min | 74.7 (12.1) | 76.1 (15.8) | 0.230 |  | 75.1 (12.7) | 74.5 (13.7) | 0.710 |
| Troponin I, mean (SD) mg/dL | 9.5 (15.0) | 11.5 (14.7) | 0.092 |  | 10.2 (15.7) | 10.9 (17.4) | 0.724 |
| CK-MB, mean (SD) mg/dL | 16.8 (38.2) | 19.7 (33.3) | 0.333 |  | 18.1 (39.1) | 16.6 (35.5) | 0.736 |
| **Depression diagnosis and treatment allocation, N (%)** |  |  |  |  |  |  |  |
| Absent depression | 553 (71.4) | 38 (19.5) | **<0.001** |  | 392(62.8) | 34 (39.1) | **<0.001** |
| Depression on escitalopram | 67 (8.7) | 60 (30.8) |  |  | 82 (13.1) | 14 (16.1) |  |
| Depression on placebo | 62 (8.0) | 66 (33.8) |  |  | 74 (11.9) | 21 (24.1) |  |
| Depression on care as usual | 92 (11.9) | 31 (15.9) |  |  | 76 (12.2) | 18 (20.7) |  |
| ^a^p-values were determined using *t*-tests or χ^2^ tests as appropriate.  ACS = acute coronary syndrome, LVEF = left ventricular ejection fraction, CK-MB = creatine kinase-MB | | | | | | | |

**Supplemental Fig. 1.** Tumor necrosis factor-α (TNF-α) serum concentrations by TNF-α two polymorphisms status.

Error bar indicates the 95% confidence interval for the mean.


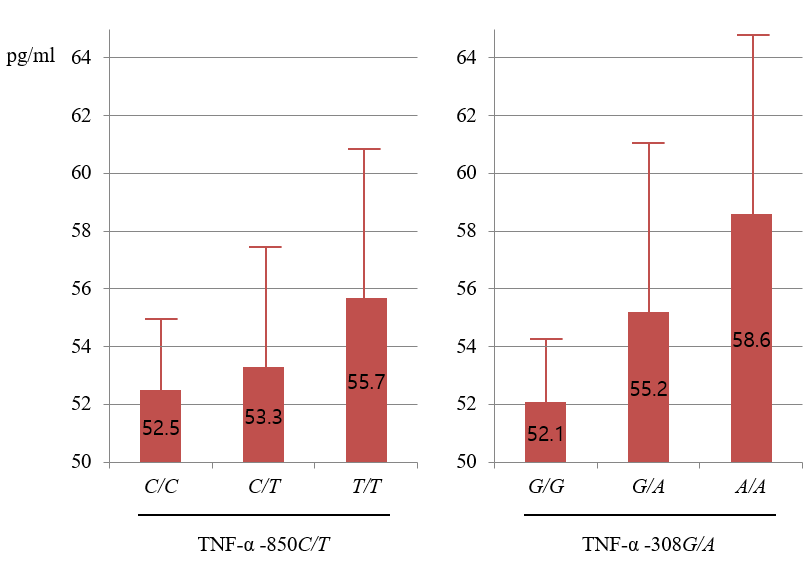

Supplement: Supplementary file 1 [file Data_Sheet_1.doc]
